# A Nomogram Based on Serum Biomarkers and Clinical Characteristics to Predict Survival in Patients With Non-Metastatic Nasopharyngeal Carcinoma

**DOI:** 10.3389/fonc.2020.594363

**Published:** 2020-12-10

**Authors:** Qing-Jie Li, Yan-Ping Mao, Rui Guo, Cheng-Long Huang, Xue-Liang Fang, Jun Ma, Ling-Long Tang, Lei Chen

**Affiliations:** Department of Radiation Oncology, Sun Yat-sen University Cancer Center, Guangzhou, China

**Keywords:** nasopharyngeal carcinoma, nomogram, serum biomarkers, prognostic prediction, survival

## Abstract

**Objective:**

This study focused on developing an effective nomogram for improving prognostication for patients with primary nasopharyngeal carcinoma (NPC) restaged according to the eighth edition of the AJCC/UICC TNM staging system.

**Methods:**

Based on data of 5,903 patients with non-metastatic NPC (primary cohort), we used Cox regression analysis to identify survival risk factors and created a nomogram. We used the nomogram to predict overall survival (OS), distant metastasis-free survival (DMFS) and disease-free survival (DFS) in the primary and independent validation (3,437 patients) cohorts. Moreover, we compared the prognostic accuracy between the 8th TNM system and the nomogram.

**Results:**

The nomogram included gender, age, T stage, N stage, Epstein–Barr virus DNA, hemoglobin, C-reactive protein, lactate dehydrogenase, and radiotherapy with/without induction or concurrent chemotherapy. In the prediction of OS, DMFS and DFS, the nomogram had significantly higher concordance index (C-index) and area under ROC curve (AUC) than the TNM system alone. Calibration curves demonstrated satisfactory agreements between nomogram-predicted and observed survival. The stratification in different groups permitted remarkable differentiation among Kaplan–Meier curves for OS, DMFS, and DFS.

**Conclusion:**

The nomogram led to a more precise prognostic prediction for NPC patients in comparison with the 8th TNM system. Therefore, it could facilitate individualized and personalized patients’ counseling and care.

## Introduction

Nasopharyngeal carcinoma (NPC), which arises from the nasopharynx epithelium, is the commonest head and neck malignant tumor in southeastern Asia and southern China (1). The risk factors for NPC contain genetic sensitivity, diet, Epstein–Barr virus infection and so on (2, 3). NPC caused 129,079 incident cases as well as triggered 72,987 deaths worldwide in 2018 (4). As for its treatment, radiotherapy is the mainstay therapy for patients with NPC. Additionally, combined chemoradiation has better efficaciousness in the therapy in advanced stage of NPC (5). Nevertheless, the survival of most NPC patients remains poor. Furthermore, though patients who were in the same TNM stage and obtained similar treatments, more than 20% of the patients showed poor effect (6), which indicated that therapy failure was partly attributed to the prognostic evaluation of the TNM staging system.

Therefore, besides trying our best to improve therapies for NPC, making prognostic evaluation more precise is also necessary for us to determine the malignant grade of NPC, and optimize treatment. The AJCC/UICC TNM staging system is the commonest prognostic factor. However, previous studies illustrated that sometimes this staging system fails to predict prognosis satisfactorily (7–9). Thus, recognizing factors related to prognosis can ameliorate the TNM staging system to predict survival of NPC patients. In recent years, an increasing number of serum markers, which can be conveniently gained, were regarded as prognostic markers for NPC patients, containing Epstein–Barr virus DNA (EBV-DNA) (10), hemoglobin (HGB) (11), albumin (ALB) (12), C-reactive protein (CRP) (13), lactate dehydrogenase (LDH) (14) and so on. These factors serve as practical biomarkers in common clinical testing.

Recently, nomograms function as new reliable tools for prognosis prediction in carcinomas (15–17). Nomograms involve some variables by analyzing their respective effects on survival and serve as convenient models to predict survival (18). Therefore, based on the data of 9,340 patients with non-metastatic NPC, we analyzed the prognostic effects of the serum factors on NPC. Besides hematological features, we also incorporated the TNM staging system and clinical factors to establish a nomogram to precisely predict overall survival (OS), distant metastasis-free survival (DMFS), and disease-free survival (DFS) of NPC patients, which can aid clinical decision making and enhance treatment effects.

## Materials and Methods

### Patients

NPC patients were divided into a primary cohort (5,903 patients, about 60% of all data in this study) and a validation cohort (3,437 patients, remaining about 40% of the data) according to the chronological order in which these patients received initial treatments. From January 2009 to June 2014, 5,903 primary NPC patients at Sun Yat-sen University Cancer Center were collected in the primary cohort. The inclusion criteria were as follows: [1] non-metastatic NPC patients confirmed by histopathology; [2] adequate clinical data and examination information; [3] no distant metastasis before or during therapies; [4] no evidence for other sources of tumor or other serious diseases. Additionally, we used the same criteria to screen 3,437 primary NPC patients from July 2014 to April 2016 at the same institution and regarded them as an independent validation cohort.

All NPC patients would receive radiotherapy with/without induction or concurrent chemotherapy. For NPC patients receiving induction chemotherapy (IC), docetaxel plus cisplatin/nedaplatin plus 5-fluorouracil, or docetaxel plus cisplatin/nedaplatin, or gemcitabine plus cisplatin/nedaplatin, or cisplatin/nedaplatin plus 5-fluorouracil was administered every 3 weeks for three cycles before radiotherapy. Concurrent chemotherapy (CC) consisted of cisplatin administered every 3 weeks for 2–3 cycles (100 mg/m^2^) or weekly until the completion of radiotherapy (40 mg/m^2^). For NPC patients with a contraindication to cisplatin, nedaplatin or carboplatin was substituted.

The patients’ gender, age, smoking, or drinking history, family history of tumor, radiotherapy with/without induction chemotherapy (IC) or concurrent chemotherapy (CC), and serological data including pretreatment (pre-) EBV-DNA levels, pre-HGB, pre-ALB, pre-CRP, and pre-LDH, were obtained from the clinical records. We restaged all patients by the eighth edition of the AJCC/UICC TNM staging system. The data of all NPC patients’ serum biomarkers and clinical characteristics were measured and collected within the two weeks before initiating treatment. The Hospital Ethics Committee at Sun Yat-sen University Cancer Center in China approved the study, which analyzed anonymous information as well as waived the demand for informed consent.

### Follow-Up

Our main endpoint was overall survival (OS), and secondary endpoints were distant metastasis-free survival (DMFS) as well as disease-free survival (DFS). Patients were followed up every three months in the first two years, every six months in the next three years, and annually thereafter until death.

### Statistical Analysis

We transformed continuous variables into categorical variables. The age was grouped into <40, 40–49, 50–59, and ≥60 years old. According to the standard of anemia, pre-HGB was grouped into <120 g/L and ≥120 g/L. The optimal cut-off values for other continuous variables were determined by maximizing Youden’s index calculated in the data of the primary cohort, which serve as the difference between sensitivity and 1-specificity in the receiver operating characteristic (ROC) curves. Based on the maximizing Youden’s index of OS in NPC patients, all cut-off values were as follows: pre-EBV-DNA levels (4,000 copies/ml), pre-ALB (45 g/L), pre-CRP (2 mg/L), and pre-LDH (180 U/L).

Variables satisfying *P <*0.05 in univariate Cox regression analyses were put into multivariable analysis. *P <*0.05 in multivariable Cox regression analyses selected independent prognostic variables of survival. The TNM staging system and therapy items were regarded as necessary prognostic variables of survival in this study. All independent or necessary prognostic factors were used to create a predictive nomogram (by the package of rms in R).

The Akaike information criterion (AIC) and concordance index (C-index) with 95% confidence intervals (CIs) for the model were calculated to assess the accuracy of the nomogram in the primary and validation cohorts. Calibration plots for OS, DMFS, and DFS at three and five years were done by comparing predicted OS, DMFS, and DFS with actual OS, DMFS, and DFS. Moreover, for comparing the nomogram with the TNM staging system, the predictive precision and discrimination of the nomogram and the TNM system were analyzed by AIC, C-index (95% CI), area under curve (AUC) of ROC curves, and decision curves.

The curves for OS, DMFS, and DFS were performed using the Kaplan–Meier method. The comparisons of survival among three risk groups were analyzed using the log-rank test.

We completed the statistical analysis by R version 3.6.1 software (http://www.r-project.org) and IBM SPSS software version 25.0 (IBM, Armonk, NY, USA). Statistical data were all two-sided, and the significant effect was determined as *P <*0.05.

## Results

### Patient Characteristics and Follow-Up

5,903 (primary cohort) and 3,437 (validation cohort) patients with NPC were found eligible for this study. The median age was 45 (range, 7–80) years old for the primary cohort and 45 (range, 6–85) years old for the independent validation cohort. The male-to-female ratio was 2.86:1 (primary cohort) and 2.57:1 (validation cohort). **Table 1** listed the comparisons between the primary cohort and validation cohort, for which patients in the validation cohort had poor N stage together with lower levels of pre-EBV-DNA, pre-HGB, pre-CRP, and pre-LDH.

**Table 1:** Comparison of the different characteristics between NPC patients in the primary and validation cohorts.

Characteristic	Number of NPC patients (%)			*P*-value
	All patients(n = 9341)	Primary cohort(n = 5904)	Validation cohort(n = 3437)	
**Gender**				0.026
Male	6,853 (73.4)	4,377 (74.1)	2,476 (72.0)	
Female	2,487 (26.6)	1,526 (25.9)	961 (28.0)	
**Age** (years old)				0.012
<40	2,939 (31.5)	1,877 (31.8)	1,061 (30.9)	
40–49	3,208 (34.3)	2,079 (35.2)	1,129 (32.8)	
50–59	2,124 (22.8)	1,292 (21.9)	833 (24.2)	
≥60	1,069 (11.4)	655 (11.1)	414 (12.1)	
**Smoking**				0.004
No	6,079 (65.1)	3,778 (64.0)	2,301 (66.9)	
Yes	3,261 (34.9)	2,125 (36.0)	1,136 (33.1)	
**Drinking**				0.569
No	8,046 (86.1)	5,076 (86.0)	2,970 (86.4)	
Yes	1,294 (13.9)	827 (14.0)	467 (13.6)	
**Family history of tumor**				0.526
No	6,867 (73.5)	4,327 (73.3)	2,540 (73.9)	
Yes	2,473 (26.5)	1,576 (26.7)	897 (26.1)	
**8th T stage**				0.269
T1	1,527 (16.3)	994 (16.8)	533 (15.5)	
T2	1,510 (16.2)	996 (16.4)	544 (15.8)	
T3	4,349 (46.6)	2,716 (46.0)	1,633 (47.5)	
T4	1,954 (20.9)	1,227 (20.8)	727 (21.2)	
**8th N stage**				<0.001
N0	1,433 (15.3)	920 (15.6)	513 (14.9)	
N1	4,711 (50.4)	3,047 (51.6)	1,664 (48.5)	
N2	2,032 (21.8)	1,271 (21.5)	761 (22.1)	
N3	1,164 (12.5)	665 (11.3)	499 (14.5)	
**pre-EBV-DNA**				<0.001
<4000 copies/ml	5,363 (57.4)	3,156 (53.5)	2,207 (64.2)	
≥4,000 copies/ml	3,977 (42.6)	2,747 (46.5)	1,230 (35.8)	
**pre-HGB**				0.001
<120 g/L	646 (6.9)	369 (6.3)	277 (8.1)	
≥120 g/L	8,694 (93.1)	5,534 (93.7)	3,160 (91.9)	
**pre-ALB**				0.502
<45 g/L	5,467 (58.5)	3,440 (58.3)	2,027 (59.0)	
≥45 g/L	3,873 (41.5)	2,463 (41.7)	1,410 (41.0)	
**pre-CRP**				0.013
<2 mg/L	5,255 (56.3)	3,264 (55.3)	1,991 (57.9)	
≥2 mg/L	4,085 (43.7)	2,639 (44.7)	1,446 (42.1)	
**pre-LDH**				<0.001
<180 U/L	5,128 (54.9)	3,087 (52.3)	2,041 (59.4)	
≥180 U/L	4,212 (45.1)	2,816 (47.7)	1,396 (40.6)	
**IC**				0.991
No	4,706 (50.4)	2,974 (50.4)	1,732 (50.4)	
Yes	4,634 (49.6)	2,929 (49.6)	1,705 (49.6)	
**CC**				0.224
No	1,932 (20.7)	1,244 (21.1)	688 (20.0)	
Yes	7,408 (79.3)	4,659 (78.9)	2,749 (80.0)	

ALB albumin; CC, concurrent chemotherapy; CRP; C-reactive protein; EBV-DNA, Epstein–Barr virus DNA (EBV-DNA), HGB, hemoglobin; IC, induction chemotherapy; LDH, lactate dehydrogenase; pre, pretreatment.

All continuous variables were changed to categorical variables. Pearson χ^2^ test was used to compute the P-value.

The median follow-up for OS, DMFS, and DFS as well as the 3- and 5-year OS, DMFS, and DFS were shown in **Table 2**.

**Table 2:** The median follow-up for OS, DMFS, and DFS as well as the 3- and 5-year OS, DMFS, and DFS.

	OS	DMFS	DFS	OS	DMFS	DFS
	primary cohort			validation cohort		
**median follow-up (month)**	76	76	74	53	53	52
**[range]**	[4–123]	[3–123]	[3–119]	[2–65]	[1–65]	[1–65]
**3-year survival (%)**	91.7	88.9	82.5	91.8	89.3	82.9
**5-year survival (%)**	84.7	86.3	77.1	87.6	87.9	79.0

DFS, disease-free survival; DMFS, distant metastasis-free survival; OS, overall survival.

### Univariate and Multivariate Cox Regression Analyses

**Figure 1:**
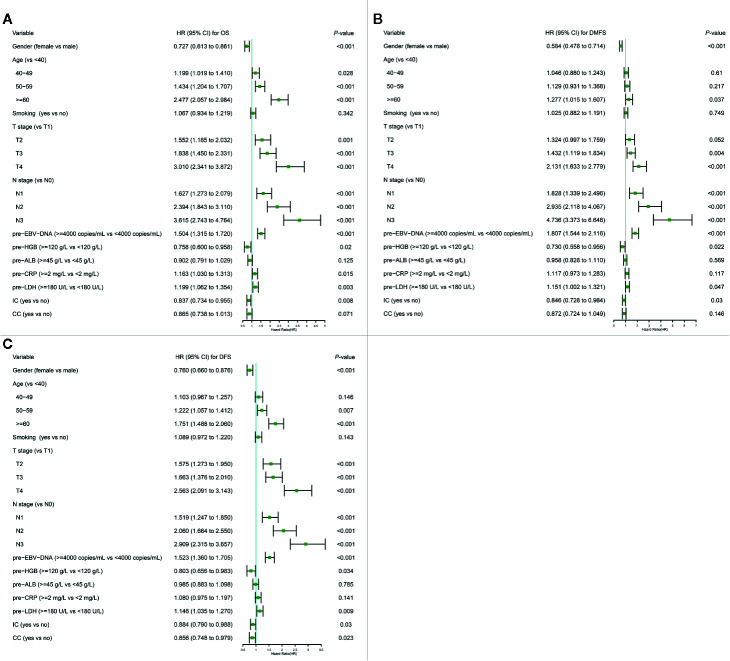
Multivariate Cox regression analysis of OS **(A)**, DMFS **(B)**, and DFS **(C)** in the primary cohort. ALB, albumin; CC, concurrent chemotherapy; CI, confidence interval; CRP, C-reactive protein; DFS, disease-free survival; DMFS, distant metastasis-free survival; EBV-DNA, Epstein–Barr virus DNA; HR, hazard ratio; HGB, hemoglobin; IC, induction chemotherapy; LDH, lactate dehydrogenase; OS, overall survival; pre-, pretreatment (pre-). Cox proportional hazard model was used to conduct Cox regression analysis.

The variables significantly related to poorer OS in univariate Cox regression analysis were gender (male); advanced age, T stage, N stage; smoking history; higher plasma pre-EBV-DNA (≥4000 copies/ml), pre-CRP (≥2 mg/L) and pre-LDH (≥180 U/L); lower pre-HGB (<120 g/L) and pre-ALB(<45 g/L); radiotherapy with induction chemotherapy (IC) (**Table 3**). Some phase III randomized trials proved that radiotherapy with concurrent chemotherapy (CC) is the standard therapy for advanced nasopharyngeal carcinoma, which remarkably ameliorates the survival of NPC patients (19, 20). In this study, radiotherapy with/without CC was an independent prognostic factor for DFS (**Figure 1C**). Thus, though radiotherapy with/without CC had a non-significant *P*-value of 0.076 for OS, we still regarded it as a necessary prognostic variable of survival in this study and put it into multivariate Cox regression analysis as well as the establishment of the nomogram. All factors above entered into multivariate Cox regression analysis. Finally, gender, age, T stage, N stage, plasma pre-EBV-DNA, pre-HGB, pre-CRP, pre-LDH, and radiotherapy with/without IC or CC were the significant prognostic factors. Detailed summaries of univariate and multivariate Cox analysis for OS, DMFS, and DFS were shown in **Table 3** and **Figure 1**.

**Table 3:** Univariate Cox regression analysis of OS, DMFS, and DFS in the primary cohort.

Variable	OS		DMFS		DFS	
	HR (95% CI)	*P*-value	HR (95% CI)	*P*-value	HR (95% CI)	*P*-value
**Gender**						
Male	Reference		Reference		Reference	
Female	0.701 (0.606 to 0.812)	<0.001	0.593 (0.497 to 0.708)	<0.001	0.735 (0.650 to 0.830)	<0.001
**Age**						
<40	Reference		Reference		Reference	
40–49	1.187 (1.011 to 1.394)	0.036	1.090 (0.869 to 1.222)	0.726	1.090 (0.957 to 1.241)	0.194
50–59	1.534 (1.294 to 1.818)	<0.001	1.184 (0.983 to 1.427)	0.075	1.290 (1.120 to 1.485)	<0.001
≥60	2.895 (2.427 to 3.451)	<0.001	1.477 (1.187 to 1.837)	<0.001	1.994 (1.709 to 2.326)	<0.001
**Smoking**						
no	Reference		Reference		Reference	
yes	1.396 (1.240 to 1.573)	<0.001	1.386 (1.210 to 1.587)	<0.001	1.348 (1.218 to 1.491)	<0.001
**Drinking**						
no	Reference		Reference		Reference	
yes	1.168 (0.993 to 1.373)	0.061	1.233 (1.029 to 1.478)	0.023	1.192 (1.039 to 1.366)	0.012
**Family history of tumor**						
no	Reference		Reference		Reference	
yes	0.918 (0.802 to 1.051)	0.216	0.936 (0.803 to 1.092)	0.402	0.913 (0.814 to 1.024)	0.121
**8th T stage**						
T1	Reference		Reference		Reference	
T2	1.887 (1.446 to 2.463)	<0.001	1.618 (1.222 to 2.141)	<0.001	1.835 (1.487 to 2.264)	<0.001
T3	2.332 (1.853 to 2.934)	<0.001	1.834 (1.444 to 2.331)	<0.001	2.001 (1.668 to 2.400)	<0.001
T4	4.076 (3.219 to 5.161)	<0.001	2.940 (2.291 to 3.773)	<0.001	3.299 (2.731 to 3.987)	<0.001
**8th N stage**						
N0	Reference		Reference		Reference	
N1	1.907 (1.505 to 2.415)	<0.001	2.232 (1.655 to 3.010)	<0.001	1.791 (1.484 to 2.161)	<0.001
N2	3.137 (2.453 to 4.013)	<0.001	4.089 (3.010 to 5.555)	<0.001	2.692 (2.207 to 3.284)	<0.001
N3	4.859 (3.767 to 6.267)	<0.001	6.886 (5.040 to 9.408)	<0.001	3.922 (3.184 to 4.832)	<0.001
**pre-EBV-DNA**						
<4,000 copies/ml	Reference		Reference		Reference	
≥4,000 copies/ml	2.266 (2.004 to 2.561)	<0.001	2.694 (2.332 to 3.112)	<0.001	2.118 (1.911 to 2.347)	<0.001
**pre-HGB**						
<120 g/L	Reference		Reference		Reference	
≥120 g/L	0.778 (0.623 to 0.971)	0.027	0.798 (0.618 to 1.029)	0.082	0.822 (0.678 to 0.997)	0.046
**pre-ALB**						
<45 g/L	Reference		Reference		Reference	
≥45 g/L	0.686 (0.606 to 0.777)	<0.001	0.810 (0.705 to 0.930)	0.003	0.809 (0.729 to 0.896)	<0.001
**pre-CRP**						
<2 mg/L	Reference		Reference		Reference	
≥2 mg/L	1.526 (1.356 to 1.718)	<0.001	1.461 (1.277 to 1.672)	<0.001	1.354 (1.226 to 1.497)	<0.001
**pre-LDH**						
<180 U/L	Reference		Reference		Reference	
≥180 U/L	1.501 (1.333 to 1.690)	<0.001	1.444 (1.261 to 1.654)	<0.001	1.381 (1.250 to 1.527)	<0.001
**IC**						
No	Reference		Reference		Reference	
Yes	1.423 (1.263 to 1.603)	<0.001	1.509 (1.316 to 1.730)	<0.001	1.408 (1.273 to 1.557)	<0.001
**CC**						
No	Reference		Reference		Reference	
Yes	1.145 (0.986 to 1.329)	0.076	1.260 (1.057 to 1.503)	0.01	1.156 (1.018 to 1.311)	0.025

CI, confidence interval; HR, hazard ratio.

Cox proportional hazard model was used to conduct Cox regression analysis.

### Establishing and Validating a Nomogram

**Figure 2:**
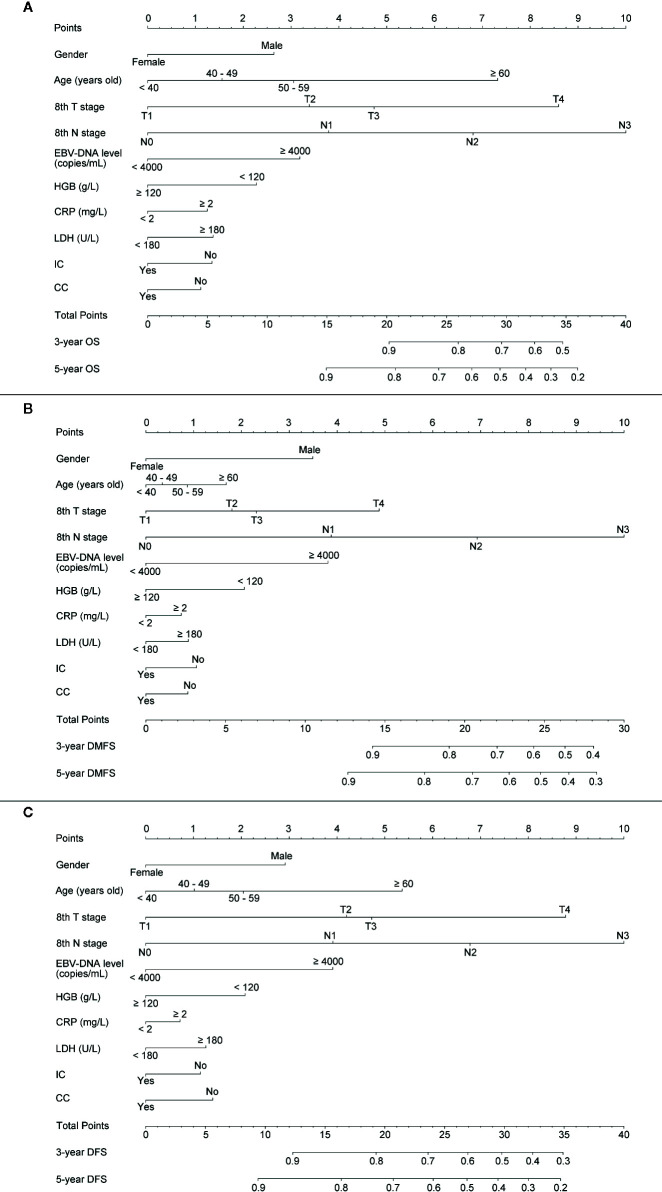
Establishing a nomogram. Nomogram was based on gender, age, T stage, N stage, plasma pre-EBV-DNA, pre-HGB, pre-CRP, pre-LDH, and radiotherapy with/without induction or concurrent chemotherapy for 3-, 5-year OS **(A)**, DMFS **(B),** and DFS **(C)** in NPC patients in the primary cohort.

For providing a clinically quantitative tool to predict OS, DMFS as well as DFS probability, a nomogram was created based on the important prognostic factors mentioned above. All factors were involved, including gender, age, 8th T stage, 8th N stage, plasma pre-EBV-DNA, pre-HGB, pre-CRP, pre-LDH, and radiotherapy with/without IC or CC. By aggregating the score of each variable and locating the total scores on the score scale, the nomogram was constructed to prognosticate 3- as well as 5-year OS, DMFS, and DFS in the primary cohort (**Figures 2A–C**).

**Figure 3:**
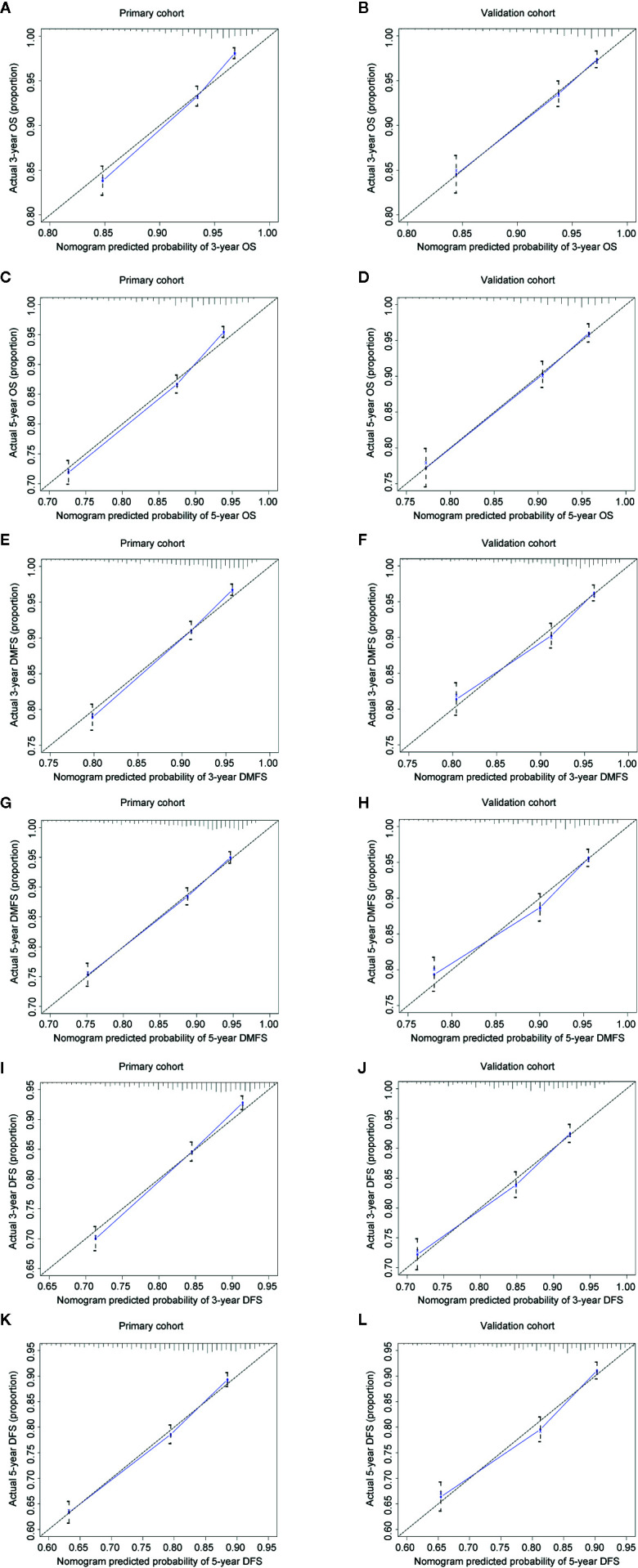
Calibration plots for 3- and 5-year OS, DMFS, and DFS in the primary **(A, C, E, G, I, K)** and validation cohorts **(B, D, F, H, J, L)**. Nomogram-predicted 3- or 5-year OS **(A–D)**, DMFS **(E–H)**, and DFS **(I-L)** were plotted on the x-axis; Kaplan–Meier calculated outcomes were plotted on the y-axis. The dashed 45-degree lines serve as the best situation indicating the predicted probabilities are equal to the actual probabilities.

The concordance index (C-index) for the nomogram to predict OS and DMFS over 0.7 in all cohorts indicated the model is satisfactory (**Table 4**). In the calibration plots, the x-axis was the prediction of OS, DMFS, or DFS computed by the nomogram, and the y-axis was the observed OS, DMFS, or DFS calculated by the Kaplan**–**Meier method. The solid line is the ideal reference line to represent the consistency between predicted survival and observed survival. Reassuringly, the calibration plots concerning the probability of three-year or five-year OS, DMFS, and DFS had remarkable correspondence between prediction and observation in all cohorts (**Figure 3**).

**Table 4:** Comparison of the eighth edition of the UICC/AJCC TNM system and the nomogram in patients with NPC.

		the 8th TNM system		the nomogram		*P*-value
		AIC	C-index (95% CI)	AIC	C-index (95% CI)	
**primary** **cohort**	**OS**	18,447.63	0.669 (0.653–0.685)	18,245.68	0.710 (0.696–0.724)	<0.01
	**DMFS**	14,253.72	0.673 (0.655–0.691)	14,150.26	0.705 (0.689–0.720)	<0.01
	**DFS**	25,825.26	0.643 (0.629–0.657)	25,672.22	0.672 (0.658–0.686)	<0.01
**validation** **cohort**	**OS**	6,072.35	0.659 (0.633–0.684)	5,977.36	0.715 (0.691–0.739)	<0.01
	**DMFS**	6,427.71	0.656 (0.631–0.681)	6,364.74	0.701 (0.677–0.725)	<0.01
	**DFS**	11,078.11	0.642 (0.622–0.662)	11,000.83	0.677 (0.657–0.697)	<0.01

AIC, Akaike information criterion.

### Comparison of the Eighth Edition of the UICC/AJCC TNM System and the Nomogram in Patients With NPC

**Figure 4:**
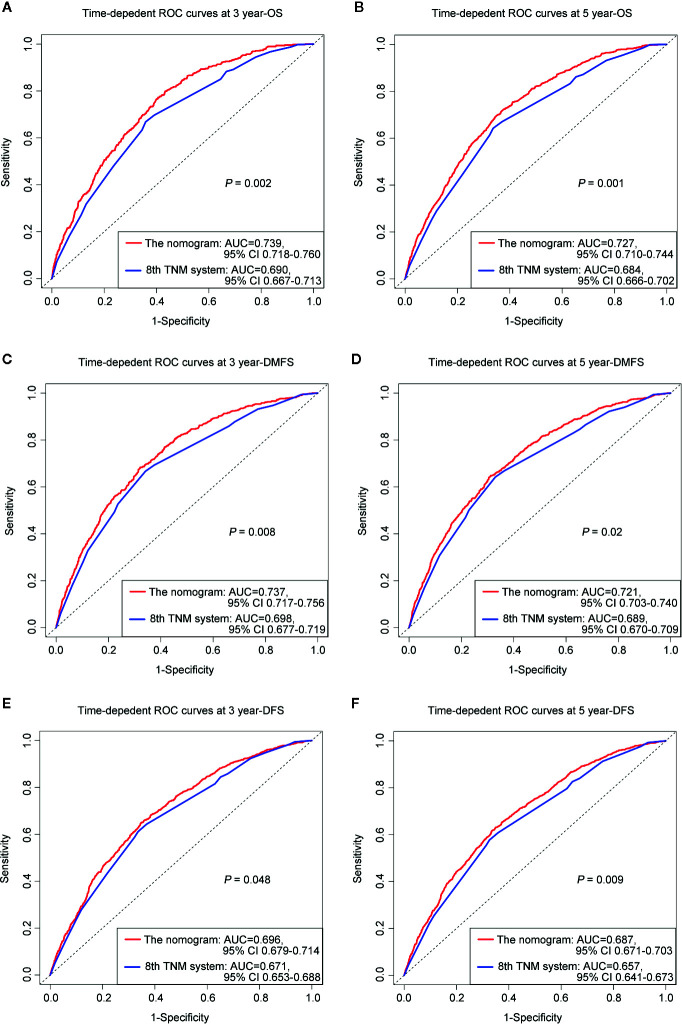
ROC curves of 3-, 5-year OS **(A, B)**, DMFS **(C, D)**, and DFS **(E, F)** for respective comparison of the nomogram with the 8th edition of the UICC/AJCC TNM system.

**Figure 5:**
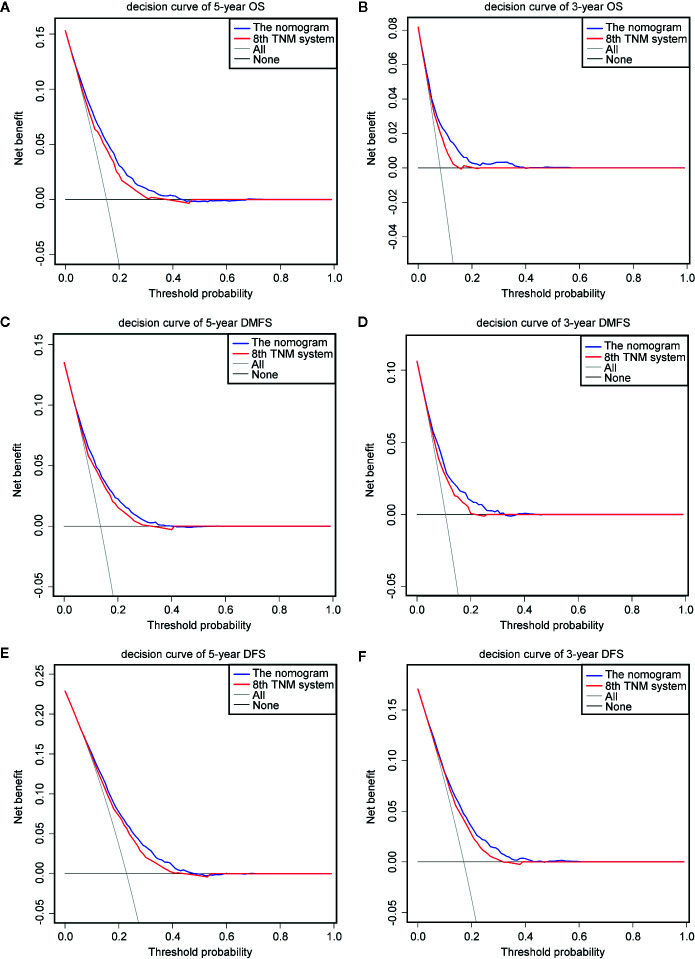
Decision curve analysis for OS, DMFS, and DFS prediction. The decision curves of the primary cohort (5-year OS, DMFS, and DFS) **(A, C, E)** and the validation cohort (3-year OS, DMFS, and DFS) **(B, D, F)**.

The prognostic accuracy of the eighth edition of the TNM system and the nomogram concerning OS, DMFS, and DFS was compared in all cohorts. As a result, the nomogram had lower Akaike information criterion (AIC) value and higher C-index than the 8th TNM system when predicting OS, DMFS, and DFS in NPC patients (**Table 4**). It revealed that the nomogram had markedly higher predictive precision and discrimination than the TNM staging system. The ROC curves of 3- and 5-year OS, DMFS, and DFS also demonstrated the better predictive function of the nomogram (**Figure 4**). Further, the decision curve indicated that the nomogram had a higher net medical benefit than the 8th TMN stage across a broader range of threshold probabilities to prognosticate OS, DMFS, and DFS in both primary (**Figures 5A, C, E**) and validation (**Figures 5B, D, F**) cohorts.

### Nomograms for Risk Stratification

**Figure 6:**
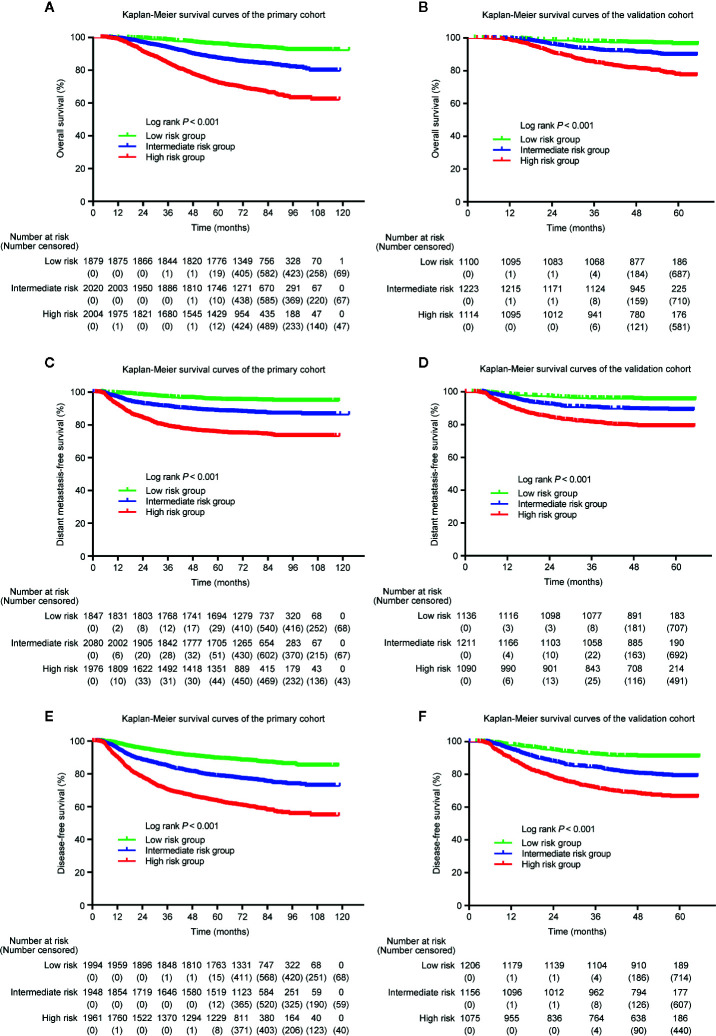
Kaplan–Meier curves of OS **(A, B)**, DMFS **(C, D)**, and DFS **(E, F)** for low-, intermediate- and high-risk groups. The stratifications of risk groups were based on the 33 and 66 percentiles of total scores in the primary **(A, C, E)** and validation cohorts **(B, D, F)**.

Because the nomogram was better than the 8th edition TNM staging system to predict OS, DMFS, and DFS, stratification was conducted based on the nomogram for OS, DMFS, and DFS. We set the cut-off values (the 33 and 66 percentiles) for the total scores calculated by the nomogram, by which the patients in the primary or validation cohorts were classified into low- [total score: <14 (OS); <11 (DMFS); <14 (DFS)], intermediate- [total score: 14**–**19 (OS); 11**–**15.5 (DMFS); 14**–**19 (DFS)] and high- [total score: > 9 (OS); >15.5 (DMFS); >19 (DFS)] risk groups. **Table 5** listed the actual 3- and 5-year OS, DMFS and DFS rates in the low-, intermediate- and high-risk groups. In the Kaplan**–**Meier OS, DMFS, and DFS curves, the risk stratification indicated a significant distinction among different risk groups (all *P*-values < 0.001; **Figure 6**).

**Table 5:** Nomograms for risk stratification.

		primary cohort			validation cohort		
		low-risk	intermediate-risk	high-risk	low-risk	intermediate-risk	high-risk
**3-year**	**OS**	98.2%	93.4%	83.9%	97.6%	92.7%	85.0%
	**DMFS**	96.9%	91.1%	79.0%	96.0%	90.3%	81.2%
	**DFS**	92.8%	84.5%	69.9%	92.0%	84.1%	71.4%
**5-year**	**OS**	95.6%	87.0%	72.0%	96.2%	95.4%	90.7%
	**DMFS**	95.3%	88.4%	75.3%	89.6%	88.9%	78.8%
	**DFS**	89.3%	78.6%	63.2%	77.0%	78.9%	66.2%

## Discussion

The 8th edition of the UICC/AJCC TNM staging system is the commonest predictor, by which NPC patients are classified based on T (tumor size), N (lymph node involvement), as well as M (distant metastasis). Nevertheless, the survival of NPC patients differs significantly in the same TNM stage (21, 22). This phenomenon may be partly due to the TNM system, which is unable to reflect the NPC patients’ prognosis completely. Thus, we need a more reliable prognostic tool to precisely predict which patients may obtain clinical benefit from more intensive therapy and avoid overtreatment.

In this study, we established and validated a nomogram for predicting OS, DMFS, and DFS in NPC patients based on serum biomarkers and clinical characteristics. The nomogram remarkably outperformed the 8th TNM system to predict 3-, 5-year OS, DMFS, and DFS, which would assist clinicians in distinguishing high-risk NPC patients as well as selecting suitable therapies.

Several serum markers serve as potential predictors of prognosis in patients with NPC. For instance, previous studies demonstrated that increased EBV-DNA level is related to local recurrence as well as distant metastasis. It is closely associated with the extent of tumor, serving as a tumor biomarker to predict survival of NPC patients (23–25). HGB is a significant marker of patients’ nutritional status. Its level reveals the state of hypoxia in tumor tissues. Some studies indicated that decreased HGB is significantly related to poorer prognosis in patients with NPC (26, 27). ALB is also an important indicator reflecting the patients’ nutritional status and has been used for prognostic assessment of patients with NPC (28). CRP, an acute-phase protein, increases quickly related to inflammation or infection (29). High level of serum CRP in NPC patients is associated with poor prognosis (30). LDH is also a prognostic marker in NPC patients, high level of which represents worse 5-year OS, DMFS, and DFS (31).

Based on these studies, the levels of pre-EBV-DNA, pre-HGB, pre-ALB, pre-CRP, and pre-LDH have been evaluated in this study, combined with gender, age, T stage, N stage, smoking, drinking history, family history of tumor, and radiotherapy with/without IC or CC. We recognized the significant prognostic factors for OS, DMFS, and DFS through univariate and multivariate Cox analyses, which included gender, age, T stage, N stage, pre-EBV-DNA, pre-HGB, pre-CRP, pre-LDH, and radiotherapy with/without IC or CC. Based on these predictive factors, the nomogram model was thus established.

The nomogram showed a significant improvement in OS, DMFS, and DFS prediction of NPC patients when compared with the TNM stage system. The model was also tested in the independent validation cohort, verifying its reliability and reproducibility. Also, according to the nomogram, we divided patients into high, intermediate, and low-risk groups, in which the high-risk group had a markedly poor OS, DMFS, and DFS.

There are four main advantages of the study. First, we had a relatively large number of patients (9,340 patients) that made the conclusion more convictive. Second, after integrating clinical features, serum markers, and the selection of therapy items into the nomogram, our nomogram can predict the survival of NPC patients more comprehensively than the TNM staging system. Third, we can get all variables included in the nomogram easily in most medical institutions, so the nomogram has wide generalizability. Last but not least, the nomogram serves as a visualized prediction tool, which may help doctors to evaluate patients with their expected survival rapidly *via* simple calculation in clinical practice. The classification of patients with different severity of the disease is beneficial to determine appropriate therapies.

To be honest, our study also has some limitations. At first, the study served as a retrospective study, which would have an inevitable selection bias. But this kind of retrospective studies is worth performing because it is significant to the design of some prospective studies. Secondly, all the cohorts involved patients at a single hospital, which may limit the applicability of our findings for patients from other geographical regions and institutions. However, the large primary cohort (more than 5,500 patients) and the independent validation cohort could largely enhance the convincingness of results.

In summary, we established and validated a nomogram to predict OS, DMFS, and DFS in NPC patients, which involved gender, age, T stage, N stage, pre-EBV-DNA, pre-HGB, pre-CRP, pre-LDH, and radiotherapy with/without IC or CC. The nomogram showed outstanding discriminative ability as well as satisfactory consistency to classify patients with NPC into low-, intermediate- and high-risk groups, which can provide helpful clues for doctors to identify the high-risk NPC patients and select suitable treatments.

## Data Availability Statement

The raw data supporting the conclusions of this article will be made available by the authors, without undue reservation.

## Ethics Statement

The studies involving human participants were reviewed and approved by The Hospital Ethics Committee at Sun Yat-sen University Cancer Center. Written informed consent from the participants’ legal guardian/next of kin was not required to participate in this study in accordance with the national legislation and the institutional requirements.

## Author Contributions

Study conception and design (L-LT, LC, and JM). Data acquisition (Y-PM, RG, C-LH, and X-LF). Data analysis and interpretation (Q-JL, Y-PM, RG, and LC). Quality control of data and algorithms (Q-JL, Y-PM, and RG). Manuscript writing (Q-JL, Y-PM, RG, and L-LT). Manuscript reviewing and approving (L-LT, LC, and JM). All authors contributed to the article and approved the submitted version.

## Funding

This study was supported by grants from the National Natural Science Foundation of China (81930072), Key-Area Research and Development Program of Guangdong Province (2019B020230002), Natural Science Foundation of Guangdong Province (2017A030312003), Health & Medical Collaborative Innovation Project of Guangzhou City, China (201803040003), Innovation Team Development Plan of the Ministry of Education (No. IRT_17R110), Overseas Expertise Introduction Project for Discipline Innovation (111 Project, B14035).

## Conflict of Interest

The authors declare that the research was conducted in the absence of any commercial or financial relationships that could be construed as a potential conflict of interest.

## Abbreviations

AIC, Akaike information criterion; ALB, albumin; AUC, area under curve; CRP, C-reactive protein; C-index, concordance index; CI, confidence interval; CC, concurrent chemotherapy; DFS, disease-free survival; DMFS, distant metastasis-free survival; EBV-DNA, Epstein–Barr virus DNA; HGB, hemoglobin; IC, induction chemotherapy; LDH, lactate dehydrogenase; NPC, nasopharyngeal carcinoma; OS, overall survival; pre-, pretreatment; ROC, receiver operating characteristic.
